# Foraging-induced craniofacial plasticity is associated with an early, robust and dynamic transcriptional response

**DOI:** 10.1098/rspb.2024.0215

**Published:** 2024-04-24

**Authors:** Emily Tetrault, Ben Aaronson, Michelle C. Gilbert, R. Craig Albertson

**Affiliations:** ^1^ Molecular and Cell Biology Graduate Program, University of Massachusetts, Amherst, MA 01003, USA; ^2^ Department of Biology, University of Massachusetts, Amherst, MA 01003, USA; ^3^ Department of Biology, Pennsylvania State University, State College, PA 16802, USA

**Keywords:** cichlid, eco-morphology, 4-bar linkage mechanism, benthic, pelagic

## Abstract

Phenotypic plasticity is the ability of a single genotype to vary its phenotype in response to the environment. Plasticity of the skeletal system in response to mechanical input is widely studied, but the timing of its transcriptional regulation is not well understood. Here, we used the cichlid feeding apparatus to examine the transcriptional dynamics of skeletal plasticity over time. Using three closely related species that vary in their ability to remodel bone and a panel of 11 genes, including well-studied skeletal differentiation markers and newly characterized environmentally sensitive genes, we examined plasticity at one, two, four and eight weeks following the onset of alternate foraging challenges. We found that the plastic species exhibited environment-specific bursts in gene expression beginning at one week, followed by a sharp decline in levels, while the species with more limited plasticity exhibited consistently low levels of gene expression. This trend held across nearly all genes, suggesting that it is a hallmark of the larger plasticity regulatory network. We conclude that plasticity of the cichlid feeding apparatus is not the result of slowly accumulating gene expression difference over time, but rather is stimulated by early bursts of environment-specific gene expression followed by a return to homeostatic levels.

## 1. Introduction

How complex phenotypes arise and are maintained over time remains an important question in evolutionary biology [1–3]. The craniofacial skeleton (e.g. skull, jaws, and other hard- and soft-tissue traits in the head) is an exquisitely complex organ whose final form relies on a balance between genetic and environmental factors [4]. Due to the explicit connection between craniofacial form, function and ecology, it is also one of the most diverse and rapidly evolving traits among vertebrates [5–7]. For example, adaptive radiations, which constitute a major source of biodiversity [8,9], are often driven by divergence in diet, and concomitant changes in foraging-related structures, including the craniofacial skeleton (e.g. [10,11]).

The search for sources of craniofacial variation have largely involved genetic, molecular and cellular mechanisms [12–17]; however, plasticity also figures prominently in shaping the craniofacial skeleton [4]. For instance, different modes of foraging can lead to distinct mechanical inputs, resulting in environment-specific remodelling and growth of the feeding apparatus (e.g. [18–21]). Notably, closely related species can vary in their ability to mount a plastic response, suggesting that this trait is heritable and can respond to selection (e.g. [3,21]). As a result, an important and ongoing area of research with respect to phenotypic plasticity includes its proximate genetic basis [22].

**Figure 1.**
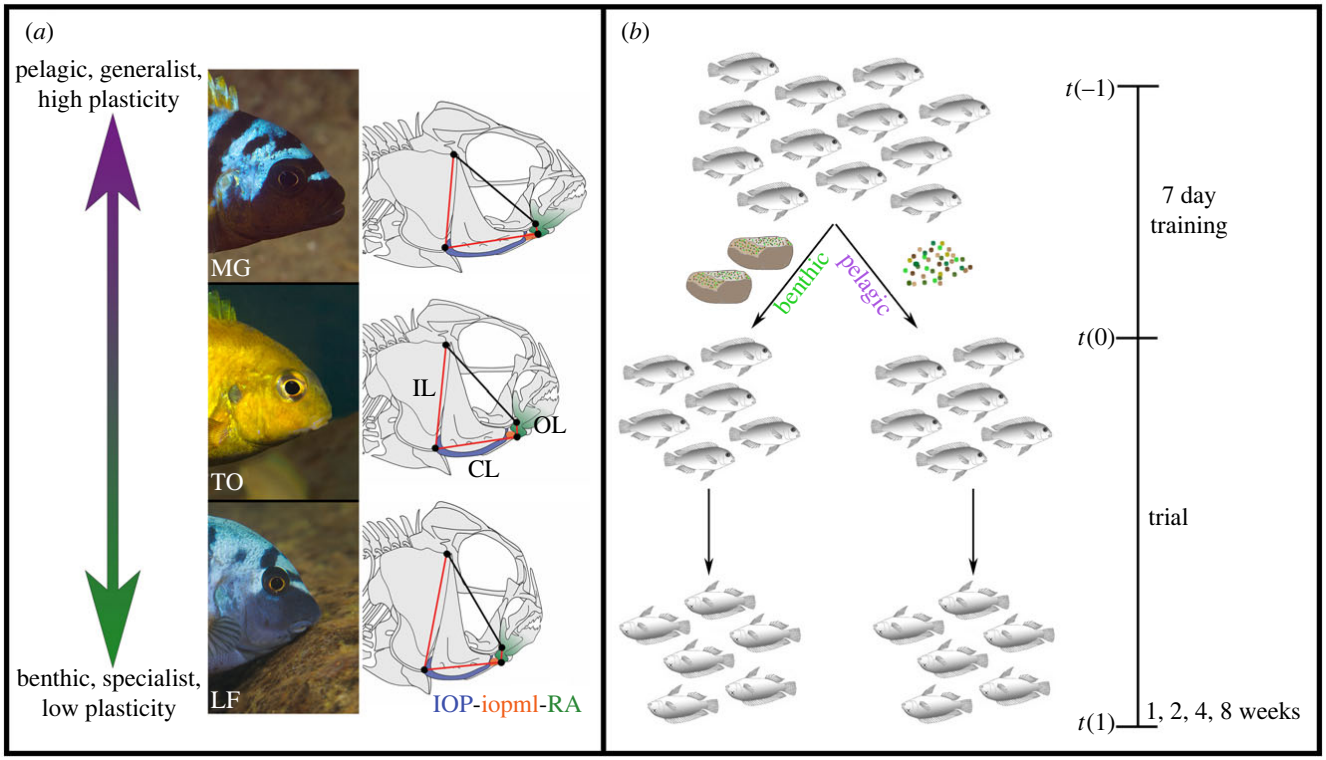
Cichlid species and experimental design. (*a*) African cichlids forage along a benthic–pelagic eco-morphological axis, and have craniofacial structures optimized for their foraging preference. For this experiment, MG, TO and LF are arrayed along this axis from pelagic generalists (MG) to benthic specialist (LF). MG should also exhibit a high degree of plasticity, whereas LF is expected to exhibit lower levels of plasticity. Species are shown along with a schematic skeletal diagram with the opercle 4-bar linkage mechanism overlaid. The three movable linkages in this system are in red, while the fixed linkage is in black. The input link (IL) is the height of the opercle bone, the coupler link (CL) is the length of the interopercle (IOP) bone plus the IOP-mandibular ligament (iopml), and the output link (OL) is the connection from the ventral tip of the retroarticular process (RA) of the lower jaw to the jaw joint. (*b*) Following a one week training period, we fed all three species either on a benthic or pelagic diet for one, two, four or eight weeks. Pelagic fish were given finely ground flake food (75% algae; 25% yolk) to force animals to suction feed from the water column, and a small amount of brine shrimp daily. Animals in the benthic environment were fed the same amount of finely ground flake food mixed with ground freeze dried brine shrimp pasted on lava rocks with food-grade agar so fish would have to apply force to scrape their food from rocks.

Recently, we took a multiomics approach to identify environmentally sensitive genes that underlie species- and environment-specific differences in jaw shapes in cichlid fishes. Focusing on functionally relevant elements of the feeding apparatus (figure 1*a*), we used overlapping RNA-seq and ATAC-seq datasets to assess differences in transcription levels and chromatin accessibility at single time-points following the onset of foraging challenges [23]. Here, we seek to characterize the expression dynamics of a subset of genes identified from that study over time, and compare them to anatomical shifts over the same time interval.

We use three African cichlid species from Lake Malawi—*Maylandia* sp*. ‘*red top Gallireya' (MG), *Tropheops* sp*. ‘*olive' (TO) and *Labeotropheus fuelleborni* (LF) (figure 1*a*)—that should vary in their plastic response to alternate benthic/pelagic foraging trails. Species from the *Maylandia* genus are generalist that feed on a range of food types, including attached algae and free-floating plankton. *Tropheops* species are benthic foragers that mainly specialize on attached algae; however, many species within this complex consume a range of food items, including plankton and detritus. Plasticity of the feeding apparatus has previously been described for *Maylandia* and *Tropheops* species at the anatomical and transcript levels [14,21,23]. By contrast, *Labeotropheus* species are extreme benthic specialists, well adapted to consume tough, filamentous, attached algae and characterized by reduced levels of craniofacial plasticity [14,21]. In this experiment, we expect to elicit a plastic response in MG and, to a lesser degree, TO*,* whereas LF should serve as a negative control, with little plasticity in anatomy or gene expression. We predict further that plasticity in gene expression will precede that in anatomy. Finally, in terms of when plasticity will manifest, there are several non-mutually exclusive possibilities. For instance, plasticity of the cichlid pharyngeal jaw and dentition have been shown to manifest gradually over several months at the transcript and anatomical levels (e.g. [19,24]). If the oral jaw apparatus follows suit, we predict that the magnitude of plasticity in gene expression will increase steadily over time. Alternatively, experiments using mammalian models have shown a transcriptional response to bone loading in less than a day (e.g. [25,26]). Under this scenario, we would predict to detect plastic gene expression at the earliest time point. Reconciling these potential outcomes, in concert with the broader body of research, will advance a better understanding of how genes and the environment interact to determine adaptive variation in jaw shape.

## 2. Methods and materials

### (a) Fish husbandry

Cichlids were housed at equal density in 40-gallon glass aquaria at approximately 28°C on a 14 h light/10 h dark cycle. Additional details are provided in electronic supplementary material. Each aquarium had constant water flow and air stones to aerate the water. Cichlid husbandry follows a protocol approved by the institutional animal care and use committee at the University of Massachusetts.

### (b) Experimental design

We used three species of African cichlid from Lake Malawi, MG (*n* = 51), TO (*n* = 61) and LF (*n* = 42) (figure 1*a*). At time 0 (figure 1*b*), we split each species into two separate tanks to isolate each treatment/species combination. Individuals in the pelagic treatment were given ground cichlid flake food to impose low amplitude, high frequency cyclic load on the oral jaw apparatus, while animals in the benthic treatment had an equal amount of ground flake food mixed with 1.5% food-grade agar and pasted over two lava rocks to impose high amplitude, low frequency static load on the jaws. Groups were given one week to train on their respective diet to ensure they learned how to forage efficiently before the start of the experiment. We sacrificed animals at four time points following the training period: one week, two weeks, four weeks and eight weeks (figure 1*b*; electronic supplementary material, table S1), allowing us to track gene expression and bone shape changes over time. We focused on the interopercle bone (IOP), the retroarticular process of the mandible (RA), and the ligament that connects the two (iopml), which represent key elements of the opercle 4-bar linkage system [27] (figure 1*a*). This ‘IOP-RA complex' was dissected from the left side of each animal and stored in Trizol at −80°C for RNA extraction.

### (c) RNA extraction and qPCR

**Figure 2.**
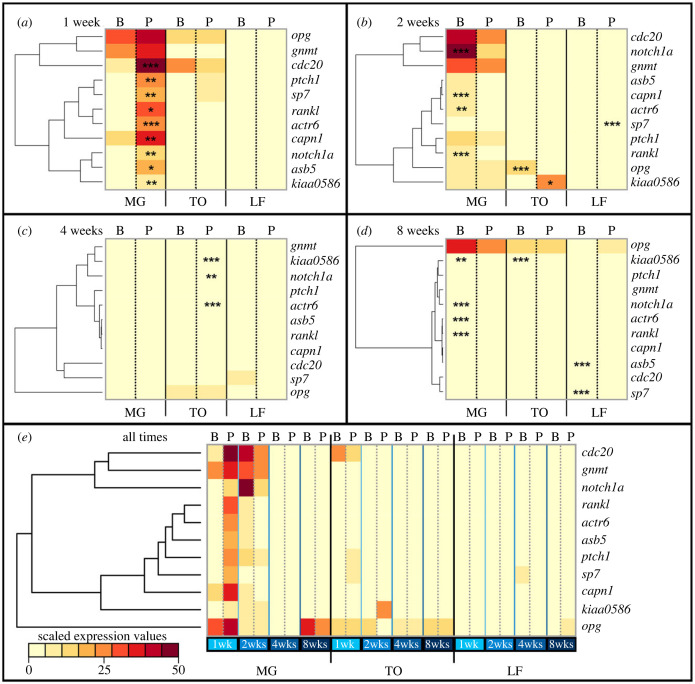
Gene expression is dynamic over time, with a marked early response in the plastic species. Gene expression is presented for all three species: MG, TO and LF. Solid lines separate species, while dotted lines separate benthic (B) and pelagic (P) treatments. Asterisks indicate significant differences in gene expression between environments, and are labelled on the treatment with higher average expression (*, ** and *** indicate significance at the *p* ≤ 0.05, *p* ≤ 0.01 and *p* ≤ 0.001 levels, respectively). Darker colours indicate higher relative expression levels compared to lighter colours (key in *e*). Because of the marked variation in relative expression levels, values were scaled as a percentage of the highest expression value across all timepoints, and then averaged within timepoints for each species/treatment (see electronic supplementary material, figure S1 for actual expression values). Positions of genes listed on the right of each heatmap are determined by the dendrogram associated with each map, and reflect covariation in gene expression. Data are presented by week (*a–d*), as well as over all time points (*e*) with species separated by solid black lines, week by solid blue lines, and environment by dotted grey lines (B, P).

While in Trizol, tissues were homogenized using a bullet blender with UFO beads (Next Advanced, Troy NY). RNA-extraction was performed using the phenol-chloroform method. Concentrations were quantified using a Nanodrop, and standardized to 70 ng µl^−1^. cDNA was prepared using the High Capacity Reverse Transcription Kit (Applied Biosystems). Levels of gene expression were measured using SYBR Green chemistry (Power SYBR Green Master Mix), and relative expression (compared to β-actin) was calculated following the 2^−ΔΔCT^ method [28]. We used *t*-tests to determine significance in expression level. We queried a panel of 11 genes including six environmentally sensitive loci from a genome-wide study (i.e. *actr6*, *asb5*, *capn1l*, *cdc20*, *gnmt*, *kiaa0586* [23]), and five additional genes based on known roles in bone development, mechanical stimulation and remodelling (*sp7*, *opg*, *rankl*, *ptch1*, *notch1a*). See primer sequences in electronic supplementary material, table S2. Because relative expression values differed by several orders of magnitude between genes, species and timepoints, we presented these data both as actual (electronic supplementary material, figure S1) and scaled values (figure 2). Additional details may be found in electronic supplementary material. All statistics were performed on values calculated using the 2^−ΔΔCT^ method. Scaled values were used to generate heatmaps in order to better illustrate the overall trends across species, treatments and time points. Here values were scaled as a percentage of the highest expression value across all timepoints, and then averaged within timepoints for each species/treatment. Heatmaps and associated dendrograms were made using Euclidean distance with the heatmap.2 function in gplots v3.1.1 [29].

### (d) Bone staining

After dissection of the left IOP-RA complex for RNA extraction, animals were stored in 4% paraformaldehyde for approximately one week at room temperature, washed with RO water and stepped through an ethanol series until 70% and stored in fresh 70% EtOH until staining. Each specimen was soaked in Alizarin Red (Sigma-Aldrich) bone stain in 70% EtOH for 24–48 h depending on the size of the animal. Excess stain was removed by further soaking each sample in 70% EtOH for 2–3 days, and stored in fresh 70% EtOH until imaging. We captured images of the right lateral surface of each specimen using a Leica M165 FC microscope with an attached Leica DFC450 camera (Leica Camera AG, Wetzlar, Germany).

### (e) Two-dimensional morphometrics and linear measures

The global craniofacial landmarking scheme consisted of 11 fixed landmarks. In addition, two curves were drawn with 11 and 5 sliding semilandmarks to outline the eye and the slope of the head, respectively (shown in figure 4, and described in electronic supplementary material). All images were digitized using StereoMorph [30]. We then ran a generalized Procrustes analysis (GPA) on landmark data. From whole shape landmark data, we quantified shape change trajectories between environments (shape ∼ species × treatment, ∼ centroid size), or over time (shape ∼ species × time, ∼centroid size) using the trajectory.analysis function in Geomorph v3.0 [31] following [32]. This function uses ANOVA to evaluate the trajectories, and calculates the differences in trajectory path and magnitude. Landmark data were subjected to 10 000 random permutations via a randomized residual permutation procedure. Each trajectory was then superimposed over the morphospace of PC1 and PC2. A test was considered significant if *p* ≤ 0.05.

Using ImageJ [33], we also took linear measures of the coupler link (CL), output link (OL) and head length, measured as the distance from the posterior edge of the opercle bone to the rostral tip of the upper jaw. Individual linkages were then plotted against head length, and, using the emtrends function in the Emmeans package [34], we generated and tested for differences in slopes. Slopes were compared between treatments within species, and between species within treatments (table 2).

## 3. Results

### (a) Dynamic gene expression by species, time and environment

We assessed gene expression at four time points, in three species, reared in two distinct foraging conditions. Several notable trends emerged from these data (table 1 and figure 2; electronic supplementary material, figure S1). First, we found that gene expression was highly dependent on genomic background (e.g. species), foraging mode and time. Nearly, every variable and interaction term had a statistically significant effect on gene expression (table 1). That expression differed by species was not surprising given their distinct craniofacial geometries; however, the widespread effects of treatment, time and interaction terms are notable, as they point to a conditional and dynamic transcriptional response to foraging conditions. For instance, we found that the overall highest levels of relative gene expression were observed at one week in MG exposed to pelagic foraging conditions, followed by benthic MG at two weeks. TO exhibited a similar, though less pronounced, pattern whereby animals exhibited higher levels of gene expression at one and two weeks, relative to later time points. Conversely, LF exhibited comparatively lower gene expression levels across all markers and timepoints. Trends in gene expression were not only observed among the environmentally sensitive genes selected from our genome-wide dataset [23], but across nearly all bone markers, suggesting that the pattern was not an artefact of choosing a biased set of genes, but rather an intrinsic feature of the larger bone regulatory network.

**Table 1.** Results of an ANOVA assessing the effects of species, treatment and time on gene expression. The specific model was Expression∼Species * Treatment * Time. Nearly every variable and interaction has a significant effect. n.s., not significant. *, ** and *** indicate significance at the *p* ≤ 0.05, *p* ≤ 0.01 and *p* ≤ 0.001 levels, respectively. • denotes 0.1 > *p* > 0.05.

	*kiaa0586*	*actr6*	*asb5*	*sp7*	*capn1l*	*gnmt*	*cdc20*	*ptch1*	*notch1a*	*opg*	*rankl*
species	*	****	****	*	****	****	****	****	****	****	****
treatment	*	**	•	***	*	n.s.	n.s.	•	•	n.s.	•
time	****	****	***	****	****	****	****	****	****	****	****
Sp:Tr	•	**	n.s.	*	*	n.s.	n.s.	•	n.s.	n.s.	n.s.
Sp:Ti	**	****	***	****	****	****	****	**	****	****	****
Tr:Ti	**	****	**	****	***	n.s.	****	****	****	•	***
Sp:Tr:Ti	**	****	**	**	****	n.s.	****	****	****	•	**

In addition to relatively high early gene expression, MG exhibited plasticity in expression at both one and two weeks. At one week, all but two genes were significantly upregulated in the pelagic compared to benthic foraging condition, and both *gnmt* and *opg* were trending higher in the pelagic environment (figure 2*a*). Notably, this pattern switched by two weeks. While fewer genes were differentially expressed between environments in MG at two weeks, those that were upregulated were from the benthic foraging environment (figure 2*b*). There was also an uptick in expression of some genes in TO at one week, but none were differentially expressed between environments (figure 2*a*). By two weeks, TO exhibited relatively higher expression of *kiaa0586* in the pelagic environment, and *opg* in the benthic environment (figure 2*b*). Thus, plastic gene expression in TO was more limited, took longer to manifest, and did not show a clear bias towards one foraging condition. In contrast to MG and TO, gene expression in LF was neither dynamic nor especially plastic in the first two weeks following the onset of foraging trails (figure 2*a,b*). The one exception was *sp7*, a core osteoblast marker (reviewed in [35]), which was expressed at a significantly higher level in fish from pelagic versus benthic treatments at two weeks (figure 2*b*). Although, given that levels of *sp7* in LF were low (max = approx. 0.003 at two weeks; electronic supplementary material, figure S1) compared to the ‘spikes' in expression of *sp7* and other genes observed in MG (greater than 0.05) and TO (greater than 0.01), it is unclear if this difference is biologically relevant.

By four weeks, we observed relatively lower levels of expression across all species and treatments (figure 2*c*), a pattern that persisted to eight weeks (figure 2*d*). Three genes were differentially expressed between environments in TO at four weeks with higher levels noted in the pelagic environment, and seven were differentially expressed between environments in all three species at eight weeks with consistently higher levels in the benthic treatment. While this consistency (i.e. higher in one treatment) is notable, overall expression levels were low compared to weeks 1 and 2, which again makes inferences about the biological relevance of these differences tenuous.

Finally, we observed a general lack of consistency in the structure of the dendrograms across timepoints in terms of both branch lengths and relative gene positions (figure 2). This was noted even for genes that encoded molecularly related proteins such as *ptch1* and *kiaa0586* (Hedgehog signalling) and *opg* and *rankl* (osteoclastogenesis), and suggests that the underlying gene network was rewired at each timepoint, underscoring the dynamic nature of the transcriptional response as animals adapted to novel foraging modes over time.

### (b) Anatomical plasticity over time

**Figure 3.**
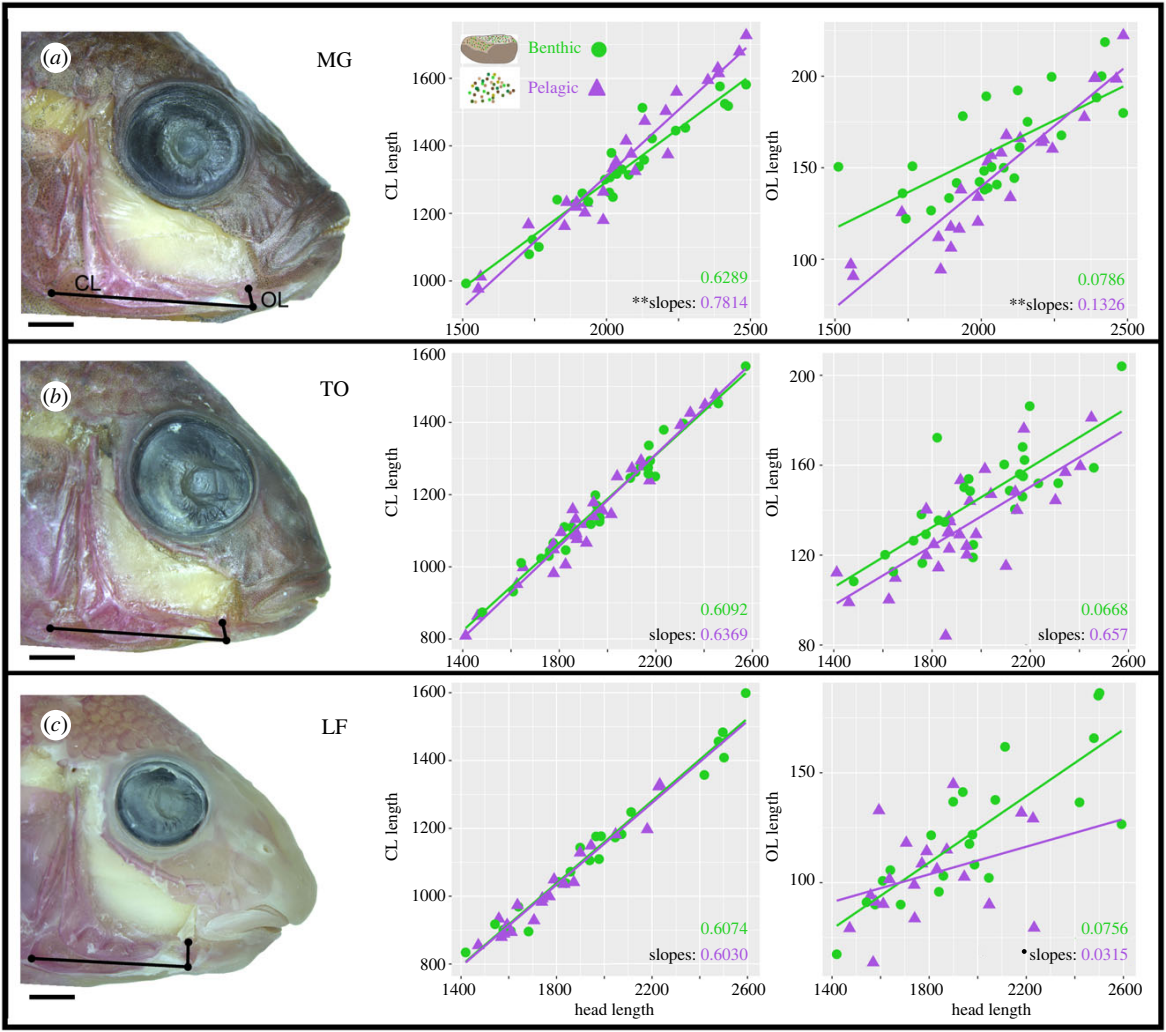
Morphological plasticity varies among species and bones. Representative images of Alizarin red stained MG (*a*), TO (*b*) and LF (*c*), with the coupler and output links (CL and OL) shown. Head length of each individual was plotted against either CL length (middle) or OL length (right). Data from the benthic treatment are represented by green circles and pelagic data are shown as purple triangles. Slopes for each species/treatment were calculated using a linear model. Divergent slopes indicate difference in growth rates, which is apparent in MG for both linkages (*a*), whereas TO have nearly identical slopes, although the *y*-intercept is greater for the OL in benthic animals (*b*). LF also have highly similar slopes for the CL, and exhibit high variation in relative OL length (*c*). Abbreviations: ** denotes *p* ≤ 0.01; • denotes 0.1 > *p* > 0.05. Scale bars equal 2.0 mm. Units are pixels, with 1 mm = 180 pixels.

We next examined growth of the same elements used to obtain expression data (dissected from the contralateral side of each fish). These included the interopercle bone (IOP) and the retroarticular process of the mandible (RA), which together represent 2/3 movable links of the opercle 4-bar linkage system: the coupler (CL) and output (OL), respectively (figure 1*a*). Growth of each element was assessed by plotting its length against overall head length and then statistically comparing slopes from a linear model for animals from different foraging environments (figure 3 and table 2). For both links, MG from the pelagic treatment exhibited significantly steeper slopes than those from the benthic treatment (figure 3*a*), consistent with our prediction that pelagic foraging should stimulate faster bone growth in this species. Pelagic foraging also drove differences in slopes between MG and the other two species, whereas slopes were statistically indistinguishable between species when reared under benthic foraging conditions (table 2). This finding is also consistent with previous work [23] showing that the pelagic environment drives species differences at the transcript level. In TO*,* slopes were nearly identical between treatments for both the CL and OL (figure 3*b*), indicating similar growth rates; however, we note that benthic foragers exhibited generally longer OLs, relative to head length, which suggests a degree of plasticity in this element. LF, likewise, exhibited nearly identical slopes between foraging treatments for the CL. A different trend was noted for the OL, where markedly different slopes were observed in LF reared in each treatment (figure 3*c*). Here animals reared in the benthic environment exhibited faster growth of the OL, and also reached larger sizes. There was also a high magnitude of variation in OL length from animals in both treatments, which resulted in slopes not being significantly different at the 0.05 level. That LF from the benthic environment grew larger underscores the idea that these are highly specialized benthic foragers that may struggle to forage using a pelagic mode [36–39]. We stress that pelagically reared LF showed no signs of being malnourished, rather they simply did not grow as fast as their benthically reared siblings (electronic supplementary material, table S1).

**Table 2.** Statistical comparison of slopes from figure 3. The top row shows for the comparison of slopes between treatments within each species. MG was the only species to exhibit a statistically significant difference in slopes, with faster growth in the pelagic versus benthic environment. Lower rows show results for the comparison between species within a treatment. n.s., not significant. *, ** and *** indicate significance at the *p* ≤ 0.05, *p* ≤ 0.01 and *p* ≤ 0.001 levels, respectively. MG grew faster for the coupler and output linkages in the pelagic environment compared to both TO and LF. Alternatively, species were indistinguishable from each other for both linkages when fed benthically.

	coupler link			output link		
	MG	TO	LF	MG	TO	LF
Ben:Pel	**	n.s.	n.s.	**	n.s.	*
	benthic	pelagic		benthic	pelagic	
MG:TO	n.s.	**		n.s.	**	
MG:LF	n.s.	**		n.s.	****	
TO:LF	n.s.	n.s.		n.s.	n.s.

**Figure 4.**
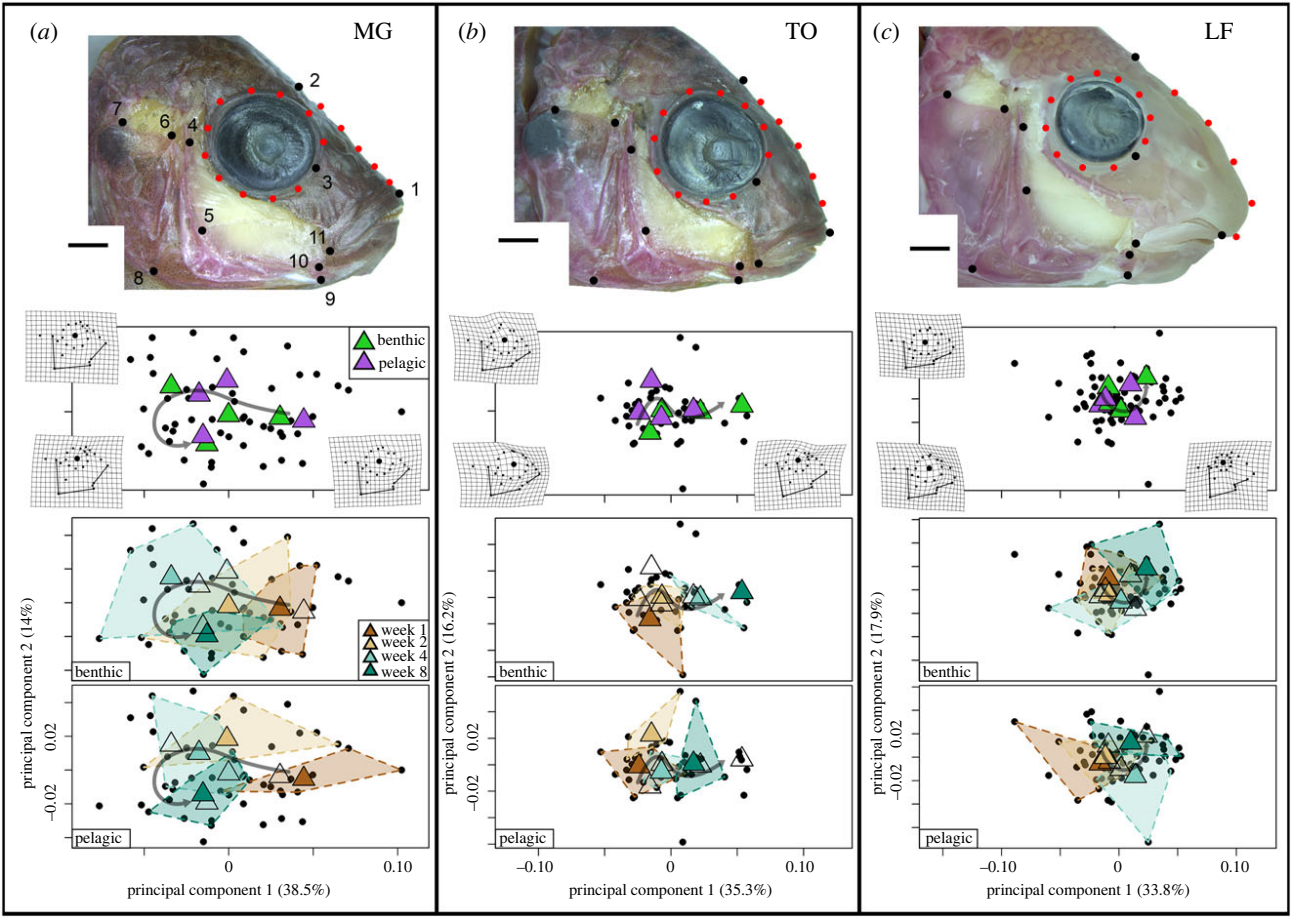
Landmarking scheme and trajectory analyses. Whole shape landmarking data for MG (*a*), TO (*b*) and LF (*c*). Fixed landmarks are denoted by black dots on the cleared and stained craniofacial profiles (see electronic supplemental material for LM descriptions). Semilandmarks around the eye and along the slope of the head are in red. Trajectory analyses are superimposed onto PC space by treatment and week for each species. Black points indicate individual animals. Coloured triangles in the top three panels depict treatment (benthic = green, pelagic = purple), or time (week 1 = dark brown, week 2 = light brown, week 4 = light teal, week 8 = dark teal) in the remaining panels. Mean path from younger to older is illustrated in a dark grey arrow on each panel. Deformation grids illustrate the outer bounds of each PC (larger grids are shown in electronic supplementary material, figure S2). Schematic of the lens, upper jaw and opercle bones are overlaid on top of the grids. Scale bars equal 2.0 mm.

We also assessed whether foraging treatments induced more global changes in craniofacial geometry over time. Using two-dimensional landmark-based geometric morphometrics, we found that craniofacial developmental trajectories were distinct between MG from each foraging treatment (trajectory shape, *p* = 0.0367), whereas they were indistinguishable between treatments in TO and LF (*p* > 0.1 for both)*.* Plotting these trajectories in PC space (figure 4) provided a visual representation of these statistics. The first notable observation was that MG from each time point occupied more of the PC plot than the other two species (figure 4*a*), whereas LF from different time points and treatments all overlapped close to the 0,0 position (figure 4*c*). TO were intermediate in terms of spread across the PC plot (figure 4*b*). We also noted slight differences in mean shapes at each stage for MG reared under benthic versus pelagic foraging conditions. In all species, ontogeny was largely captured by PC1, which characterized variation in eye size, eye position, relative head length and craniofacial profile. In addition, four- and eight-week MG were distinguished along PC2, which also captured variation in relative eye size and head length. Taken together our anatomical analyses show that MG are the most dynamic and plastic in terms of specific, functionally relevant bone lengths, as well as more global aspects of craniofacial geometry.

## 4. Discussion

### (a) The evolution, genetic basis and plasticity of a dynamic functional system

Mechanical engineering systems, including 4-bar linkages, have long been important to the fields of evolutionary and functional morphology, where they have been used to model the kinematics of fish feeding [27], avian flight [40,41], and the raptorial appendage of mantis shrimp [42]. Not only do such principles provide a way to predict function from form, but they can also reveal aspects of morphology that may be more constrained by physics than others. For instance, the evolution of kinematic transmission in both the fish opercle 4-bar system and the stomatopod raptorial appendage appears to be disproportionately driven by variability in the output linkage [43]. In other words, for fishes, changing the length of the RA may provide a more efficient means for selection to alter feeding kinematics than changes to other elements.

We have long sought to better understand the genetic basis, development and evolution of the opercle 4-bar linkage system in cichlids [44,45]. This functional complex of bones and ligaments helps to drive lower jaw depression [27], varies between species in a way that predicts foraging niche adaptation [12,44,45], and has been shown to be plastic when animals are reared under distinct foraging/mechanical conditions [21,45]. Results from the current study complement and expand this previous work.

**Figure 5.**
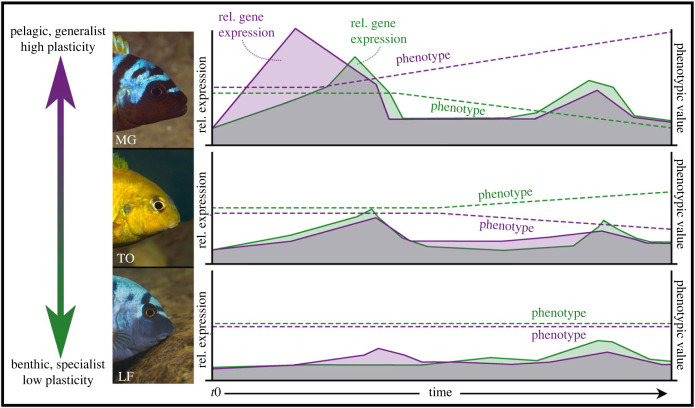
Proposed model of the transcriptional regulation of phenotypic plasticity. Species are arrayed along the same axis as in figure 1. To the right are schematic graphs summarizing the genetic and anatomical data from this study. Solid purple and green areas represent relative transcript levels over time in pelagic and benthic foraging environments, respectively. The *x*-axis is time, with t0 representing the beginning of the foraging trails. Dotted lines represent the phenotypic response in each environment, with the same colour scheme. ‘Phenotypic value' is a catch-all term that may refer to bone shape, rates of bone matrix deposition, or relative growth of a bone (e.g. [14,21]; figure 3). *Maylandia* species are generalist foragers that show a high magnitude of craniofacial plasticity, with particular sensitivity to pelagic foraging. This sensitivity is underlain by an early burst of expression, followed by a phenotypic response that continues in spite of expression values returning to base-line levels. *Labeotropheus* species are extreme benthic specialist with little sensitivity to the environment with respect to gene expression or anatomy. *Tropheops* species are also benthic foragers, but consume a more diverse diet than *Labeotropheus*, and show intermediate patterns of transcript and anatomical sensitivities. Thus, our data suggest that phenotypic plasticity is not due to the gradual accumulation of gene expression differences between environments, but rather to an early burst of environmentally sensitive gene expression. Moreover, higher magnitudes of plasticity are associated with greater and/or earlier bursts of expression.

First, we document a high degree of plasticity in the generalist forager, MG, insofar as exhibiting early and more extensive gene expression differences between foraging treatments, as well as differences in growth trajectories at the anatomical level (figure 5). These results are consistent with previously published data showing greater rates of bone deposition in *Maylandia zebra* compared to *Tropheops* sp. *‘*red cheek' (closely related and ecologically similar congeners of MG and TO) [21,23]. Also similar to previous work [14,21], we show that the specialized benthic foraging taxon, LF*,* exhibits limited anatomical plasticity and consistently low gene expression across treatments over time. Relatively low gene expression in LF may seem surprising given that this species develops the most robust craniofacial skeleton of the three species, and previous work has documented higher levels of expression of various bone markers in LF (e.g. [12,14,46,47]). This discrepancy is resolved if we consider the previous body of research in the context of homeostatic levels of expression—e.g. without an environmental stimulus—which appears to be approximated by our later time points. For example, by the four-week time point expression levels of the core bone marker, *sp7*, measures higher in LF— e.g. 0.0031 (s.e. 0.0003) under benthic foraging conditions and 0.0016 (s.e. 0.0001) under pelagic conditions—compared to MG – e.g. 0.0003 (s.e. 4.49 × 10^−05^) under benthic conditions and 0.0006 (s.e. 9.10 × 10^−05^) under pelagic conditions. Thus, in spite of the fact that animals are continuing to foraging in alternate environments, the gene regulatory system seems to be returning to more of a steady state by four weeks. There is a hint of plasticity in LF in the length of the OL/RA, which is also contrary to expectations, but consistent with a previous genetic mapping study that found that the LF allele at a locus associated with variation in RA length was sensitive to foraging treatments [48]. Placed within the larger body of research, we suggest that these cichlid genera reside at different points along a plasticity axis, with *Maylandia* exhibiting the highest magnitude of plasticity across craniofacial bones, *Labeotropheus* exhibiting limited plasticity and *Tropheops* species residing somewhere in the middle [14,21,23,48].

### (b) The importance of pelagic foraging in driving plasticity and adaptive radiations

Another notable consensus from this body of research is that pelagic foraging appears to be driving plasticity, particularly within *Maylandia*. In previous work, this was seen as greater rates of bone matrix deposition and higher relative expression of genes associated with the Hedgehog (Hh) signal transduction pathway when *M. zebra* individuals were reared under pelagic foraging conditions [21]. In a recent genome-wide analysis, nearly 4× as many genes were differentially expressed between *M. zebra* and *T.* sp*. ‘*red cheek' when animals were reared in pelagic (3761 DEG) versus benthic (984 DEG) foraging environments [23]. Here, we showed that anatomical plasticity in MG was associated with an early and dramatic burst in gene expression, particularly in individuals required to forage with a pelagic mode (figure 2). This may seem counterintuitive as bone mechanosensing is almost always associated with and studied in the context of high static loading of bone [19,26,49,50], which is more closely approximated by benthic foraging. We suggest that pelagic foraging challenges the feeding apparatus in a different way—e.g. through the (presumably) low amplitude, but high frequency action of opening and closing the oral jaws while suction feeding. In several independent experiments, we have now shown that this action can have a more pronounced effect on oral jaw bones at the transcript and tissue level, compared to biting/scraping food from rocks [21,23].

In the realm of freshwater ecology and evolution, the benthic habitat of lakes has long been recognized as an important driver of trophic segregation in fishes owing to a high level of heterogeneity [51,52]; however, the vast open-water pelagic zone may also be considered novel when ancestrally riverine populations invade large lakes, the first step in many fish adaptive radiations [11,53–56]. Plankton drives energy flow in the pelagic habitat [52], and deep lakes support a greater abundance of plankton than rivers (e.g. [57]). Adaptations associated with locating (e.g. UV vision [58]) and foraging on (e.g. suction feeding) plankton are required for fishes to successfully invade this niche. Suction feeding involves several unique musculoskeletal and kinematic features relative to other modes of foraging [59,60]; however, the ‘modulatory multiplicity’ that characterizes cichlid feeding mechanisms [61], combined with plasticity of the underlying craniofacial bones [21,62], positions cichlids to efficiently exploit (in the near term) and adapt (in the long term) to this trophic niche. *Maylandia* species appear to exemplify this process.

### (c) Insights into the proximate molecular basis of craniofacial plasticity

Our data show that craniofacial plasticity is associated with early bursts in expression of environmentally sensitive genes (figure 5), and the candidate genes involved offer insights into the molecular/cellular mechanisms that may connect plasticity at the transcript and anatomical levels. For instance, Kiaa0586 is required for primary cilium formation, as without this protein cilia axonemes fail to form [63]. Primary cilia are both mechanosensors and hubs of signal transduction pathways, including Hh signalling [64]. The Hh pathway is broadly critical for bone formation and specifically involved in shaping the craniofacial skeleton [65]. Accordingly, *kiaa0586* is required for both proper Hh signalling and craniofacial development [66,67]. We found that *kiaa0586* is plastic in both MG and TO at multiple timepoints, and that the Hh target, *ptch1*, is plastic in MG at one week. Hh signalling can regulate bone development in a number of ways, including cell-cycle regulation and differentiation of progenitor cells [68–71]. Our previous study using the same tissues implicated cell-cycle regulation [23], and both the cell-cycle marker, *cdc20,* and the bone differentiation marker, *sp7*, are differentially expressed at one week in MG (figure 2*a*). It is therefore possible that Hh signalling is leading to accelerated bone growth in pelagic MG through both cell division and differentiation. Further experiments looking at these cell behaviours immunohistochemically over time would be informative.

Some genes were plastic in multiple species but at different times, suggesting that the effects are time- and species-dependent. For example, *actr6* encodes an evolutionarily conserved actin-related protein that has been shown to localize to the nucleus and interact with heterochromatin proteins [72]. Actr6 is predicted to be a member of the SWR1 chromatin remodelling complex, supporting both its structure and function [73]. Notably, SWR1 can act as both a repressor and activator of transcription depending on the context. In plants, for instance, Actr6 has a negative effect on basal level transcription, but it has a positive effect on environmentally induced transcription [74]. Here, *actr6* expression was plastic in MG at weeks 1, 2 and 8 wks (figure 2*a,b,d*; electronic supplementary material, figure S1b and table S3), and in TO at four weeks (figure 2*c*; electronic supplementary material, figure S1b and table S3). In both species*, actr6* was initially expressed at a higher level in the pelagic environment; however, in MG it was expressed at a higher level in benthic animals at two and eight weeks (figure 2*a,b,d*; electronic supplementary material, figure S1b and table S3). It is tempting to speculate that the higher initial expression of *actr6* under pelagic foraging conditions represents an ‘induced' condition associated with transcriptional activation and accelerated bone growth, whereas the higher expression observed later in benthic MG represents the basal condition and transcriptional repression. Either way, the potential changes in chromatin state associated with differential *actr6* expression would be another interesting line of future inquiry.

While we did not find any consistent clustering of genes across time points (figure 2), *opg* remained somewhat of an outlier, especially at later time points and in the combined dataset (figure 2*c–e*), indicating that this gene is acting differently than the others in the dataset. For example, contrary to all other markers, *opg* did not decrease to its lowest level at week 8, and instead exhibited relatively high levels of expression at week 8 across all three species (figure 2*e*; electronic supplementary material, figure S1j and table S3). In the context of bone remodelling, OPG is a negative regulator of osteoclastogenesis. It is secreted from osteoblasts, along with RANKL, and while RANKL binds to its receptor, RANK, on the surface of osteoclast progenitors to promote differentiation, OPG binds to RANKL, preventing the activation of RANK. The ratio of RANKL:OPG is therefore considered an indicator of bone remodelling activity [75–77]. In LF and TO, this ratio remained consistently low over time, suggesting modest remodelling activity. Alternatively, the RANKL/OPG ratio in MG was higher, particularly at weeks 1 and 2, which suggests that not only is more bone being deposited at these time points (e.g. high early expression of *sp7*), but that bone is being remodelled to accommodate environment-specific bone architectures. That *opg* expression peaks again in MG at 8 wks, while *rankl* decreases in expression (i.e. decreasing the RANKL/OPG ratio), may suggest decreased remodelling activity and a shift to more isometric growth at this time point.

### (d) Conclusion: transcriptional dynamics of craniofacial plasticity

We took a set of marker genes with previously characterized roles in bone formation/remodelling, combined with a set previously shown to be environmentally sensitive [23], and characterized expression patterns at multiple time points following the onset of foraging challenges. Results were markedly consistent across genes, and showed that anatomical plasticity is foreshadowed by an early burst of environmentally sensitive expression, followed by a return to (presumably) baseline levels. The species that exhibited the earliest and highest relative transcript levels (MG) also exhibited the greatest phenotypic plasticity, whereas the species with no conspicuous spike in expression levels possesses a feeding structure known to be relatively robust to alternate foraging modes (LF). Based on these data (and the wider literature), we propose a model of plasticity (figure 5), whereby (i) morphological change requires an early burst of expression, and (ii) the earlier and/or more pronounced the transcriptional response, the greater the magnitude of morphological change.

Because plasticity at the anatomical level is thought to manifest gradually over time, when examining its underlying genetic basis, most studies have focused on timepoints weeks or months after the start of the experiment [19,23,24,78,79]. By contrast, our data are more in line with bio-medical research, and show that the transcriptional response to mechanical stimuli occurs in a matter of days, not weeks or months. In fact, our results suggest that important expression dynamics may be occurring at even earlier time points than those examined here.

All in all, there is still much to learn about phenotypic plasticity. Our work reveals new answers, but also new complexities. Future research should continue to drive at the molecular regulation of plasticity, how it may be affected by different environmental stimuli, and how it changes over time. At the phenotypic-level, important outstanding questions include whether plastic changes are reversible. In other words, what might happen to the phenotype if a population of fish were made to forage pelagically for two months, and then switched to a benthic mode for another two months? Further, what is the nature of the phenotypic response over time? Is it gradual and linear with respect to growth, or does it exhibit more complex, nonlinear growth? This question remains largely outstanding, particularly for skeletal plasticity, as most studies focus on a single timepoint in order to plot reaction norms (but see [18,80]). Given the nonlinear nature of our expression data, there is the possibility that the phenotypic response may be more complicated than previously assumed.

## Data Availability

Data used in this study may be found at https://doi.org/10.5061/dryad.0gb5mkm8f [81]. Supplementary material is available online [82].
